# Nitrogen Fixed By Cyanobacteria Is Utilized By Deposit-Feeders

**DOI:** 10.1371/journal.pone.0104460

**Published:** 2014-08-08

**Authors:** Agnes M. L. Karlson, Elena Gorokhova, Ragnar Elmgren

**Affiliations:** 1 Department of Applied Environmental Science, Stockholm University, Stockholm, Sweden; 2 Department of Ecology, Environment and Plant Sciences, Stockholm University, Stockholm, Sweden; Pennsylvania State University, United States of America

## Abstract

Benthic communities below the photic zone depend for food on allochthonous organic matter derived from seasonal phytoplankton blooms. In the Baltic Sea, the spring diatom bloom is considered the most important input of organic matter, whereas the contribution of the summer bloom dominated by diazotrophic cyanobacteria is less understood. The possible increase in cyanobacteria blooms as a consequence of eutrophication and climate change calls for evaluation of cyanobacteria effects on benthic community functioning and productivity. Here, we examine utilization of cyanobacterial nitrogen by deposit-feeding benthic macrofauna following a cyanobacteria bloom at three stations during two consecutive years and link these changes to isotopic niche and variations in body condition (assayed as C:N ratio) of the animals. Since nitrogen-fixing cyanobacteria have δ^15^N close to -2‰, we expected the δ^15^N in the deposit-feeders to decrease after the bloom if their assimilation of cyanobacteria-derived nitrogen was substantial. We also expected the settled cyanobacteria with their associated microheterotrophic community and relatively high nitrogen content to increase the isotopic niche area, trophic diversity and dietary divergence between individuals (estimated as the nearest neighbour distance) in the benthic fauna after the bloom. The three surface-feeding species (*Monoporeia affinis, Macoma balthica* and *Marenzelleria arctia*) showed significantly lower δ^15^N values after the bloom, while the sub-surface feeder *Pontoporeia femorata* did not. The effect of the bloom on isotopic niche varied greatly between stations; populations which increased niche area after the bloom had better body condition than populations with reduced niche, regardless of species. Thus, cyanobacterial nitrogen is efficiently integrated into the benthic food webs in the Baltic, with likely consequences for their functioning, secondary production, transfer efficiency, trophic interactions, and intra- and interspecific competition.

## Introduction

Temporal changes in trophic interactions can be considerable, particularly in temperate aquatic environments, where primary production is strongly pulsed. In these systems, growth and population dynamics of deposit-feeders is tightly coupled to sedimentation of algal blooms [1]–[5]. Consequently, deposit-feeder growth is affected by the quantity, quality and species composition of settling bloom material [6],[7]. Short-term labeling experiments in the field [8] and laboratory [9],[10],[11], show that some species respond rapidly to the freshly deposited bloom material, whereas others rely on old organic matter and associated microflora [12]. Little is known, however, about the effects of a bloom event across different levels of biological organization, or the mechanisms of the observed responses.

Both phytoplankton and benthic communities in the Baltic Sea have recently undergone large-scale changes due to a combination of eutrophication, biological invasions, and climate change. The biomass of the spring bloom, which is the most important input of high quality organic matter to the sub-thermocline soft-bottom community [13],[4],[5], has decreased, while magnitude of the summer bloom of cyanobacteria has increased [14],[15]. Although deposit-feeders in experiments consume cyanobacteria [16],[10],[11], the diatoms largely constituting the spring bloom are superior in supporting growth of benthic animals, probably because the cyanobacteria contain toxins, have low content of essential fatty acids [7],[17] and lack sterols [18]. Cyanobacteria are, however, rich in nitrogen and phosphorous [19], amino acids [20] and vitamins [21], and could, therefore, be a complementary and, perhaps, crucial food source when the high quality spring bloom input to the sediments has been exhausted. In copepods, positive effects on recruitment were observed when cyanobacteria were offered as a supplementary food source [22]. Also, bivalves performed better if fed cyanobacteria than when food deprived [23]. Moreover, cyanobacteria partially processed by, for example, clams were found to become more palatable and nutritiously upgraded for amphipods [23], thus facilitating usage of cyanobacteria-derived organic matter by benthic communities. Whether settled cyanobacteria could be eaten when relatively fresh or only after some decomposition remains, however, unclear and may differ among species.

Stable isotopes are commonly used as tracers when studying trophic relationships. They provide time-integrated information on diet and habitat use [24],[25],[26]. Ratios of ^15^N to ^14^N (expressed as δ^15^N) exhibit stepwise enrichment with each trophic transfer, whereas ratios of carbon isotopes (δ^13^C) change little with trophic transfer and can be used to determine ultimate sources of the dietary carbon. The isotopic composition of primary producers may vary over a season and influence the isotopic composition of primary consumers and predators [27],[28]. In the Baltic Sea, the summer bloom dominated by filamentous, nitrogen-fixing cyanobacteria has a characteristically depleted δ^15^N signal of about −2‰ [27],[11] that is mirrored in zooplankton [27] and littoral consumers [28],[29], and can, therefore, be used as a tracer of dietary nitrogen. Phytoplankton δ^13^C usually becomes more enriched later in the season, due to discrimination against the heavy isotope [30], but diazotrophic cyanobacteria commonly are more depleted in δ^13^C than other phytoplankton [27]. A depleted δ^13^C-signal has therefore been proposed as a tracer of cyanobacterial input in benthos [31], although the signal is not strong and might be confounded by habitat-derived differences in δ^13^C and carbon processing by microbial communities [32].

Stable isotopes can also be used to quantify subtle changes in community- or population-level trophic structure [33],[34] by quantifying distances between relative positions of individuals in the δ^13^C- δ^15^N bi-plot space; for example, the distance between the most enriched and most depleted individual (or species) in δ^13^C is the niche width of the group (population or community) with regard to the carbon sources. Recently, Tecchio et al. [35] found that trophic niche width in benthos was positively correlated with microplankton biomass in the surface water. In this context, the settled cyanobacteria bloom material with its associated microheterotrophic community would provide a diverse nutrient source to benthic communities, which could be reflected in a wider isotopic niche after the bloom. Moreover, the additional resources and the diversification of the diet could decrease both intra- and interspecific food competition in these food-limited communities [36]. Thus, access to cyanobacteria-derived material during summer could improve growth conditions for benthic consumers.

This study uses stable carbon and nitrogen isotope data from macrobenthic communities sampled at several stations during the summer season to test four interrelated hypotheses addressing incorporation of cyanobacteria-derived nitrogen and carbon in the benthic food web and the importance of cyanobacterial blooms for feeding and nutritional condition of the deposit-feeders. The tested hypotheses were:

1. In deposit-feeders, δ^15^N values will become more depleted after the cyanobacteria bloom, reflecting uptake of diazotrophic nitrogen. In addition, their δ^13^C will be also more depleted after the bloom, since cyanobacteria tend to have more depleted δ^13^C than other phytoplankton.
2. After the bloom, the isotope niche area will increase in deposit-feeders because microheterotrophic communities supported by the bloom will become more abundant and diverse, which will increase and diversify the food available to consumers.
3. Increased isotope niche-area will be positively related to the body condition of the animals, reflecting complementarity in the food sources, higher resource availability and lower intra- and interspecific competition.
4. A decreased niche overlap among the species will be observed after the bloom, due to resource diversification and differential utilization of the bloom material by co-occurring competitors. This, together with the species-specific niche increase will widen the isotope niche of the total community.

Our results show that cyanobacterial nitrogen is utilized by deposit-feeders, and indicates that the settling bloom material induces trophic changes in the community.

## Methods

### Sampling And Test Species

**Figure 1:**
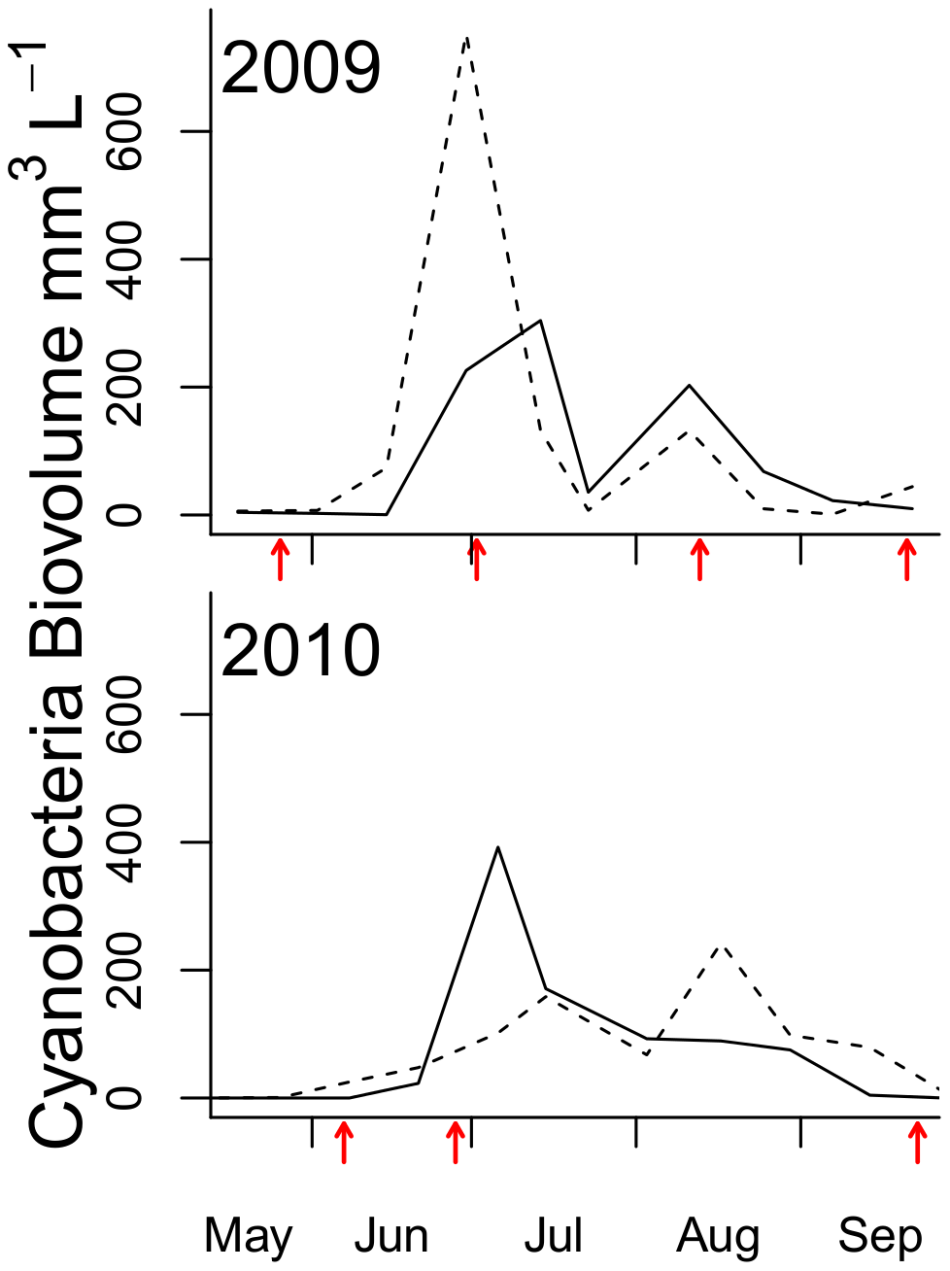
Sampling occasions in relation bloom. Cyanobacteria bloom development in 2009 and 2010 at stn B1 (near sampling stations Håldämman and Uttervik), dotted line, and stn H3 (close to stn Mörkö), solid line. The X-axis is scaled for Julian days and benthos sampling dates are indicated by arrows. See text for the description of bloom composition. In 2010, stn Mörkö was sampled only in late June and September.

We sampled sediment and deposit-feeding macrofauna at three coastal stations (stn) in the north-western Baltic proper (Figure 1); Håldämman (30 m bottom depth, 58°49′18 N, 17°34′58 E), Uttervik (20 m, 58°50′58 N, 17°32′77 E) and Mörkö (23 m, 58°54′13 N, 17°42′45 E). The sampling was scheduled around the cyanobacteria blooms in 2009 (May 26, June 2, August 13 and September 21) and 2010 (June 7, June 28 and- September 23). Stations Håldämman and Uttervik are located close (1 and 5 km, respectively) to the phytoplankton monitoring stn B1 (58°48′28 N, 17°37′60 E; Swedish National Marine Monitoring Program, data publicly available from www.smhi.se) and stn Mörkö right by the phytoplankton stn H3 (58°56′04 N, 17°43′81 E, Himmerfjärden Eutrophication study), 14 km from stn B1. No specific permission is required for sampling invertebrates in the Baltic Sea and the field studies did not involve any endangered or protected species. Figure 1 shows sampling schedule and cyanobacteria bloom development at both monitoring stations in 2009 and 2010.

**Figure 2:**
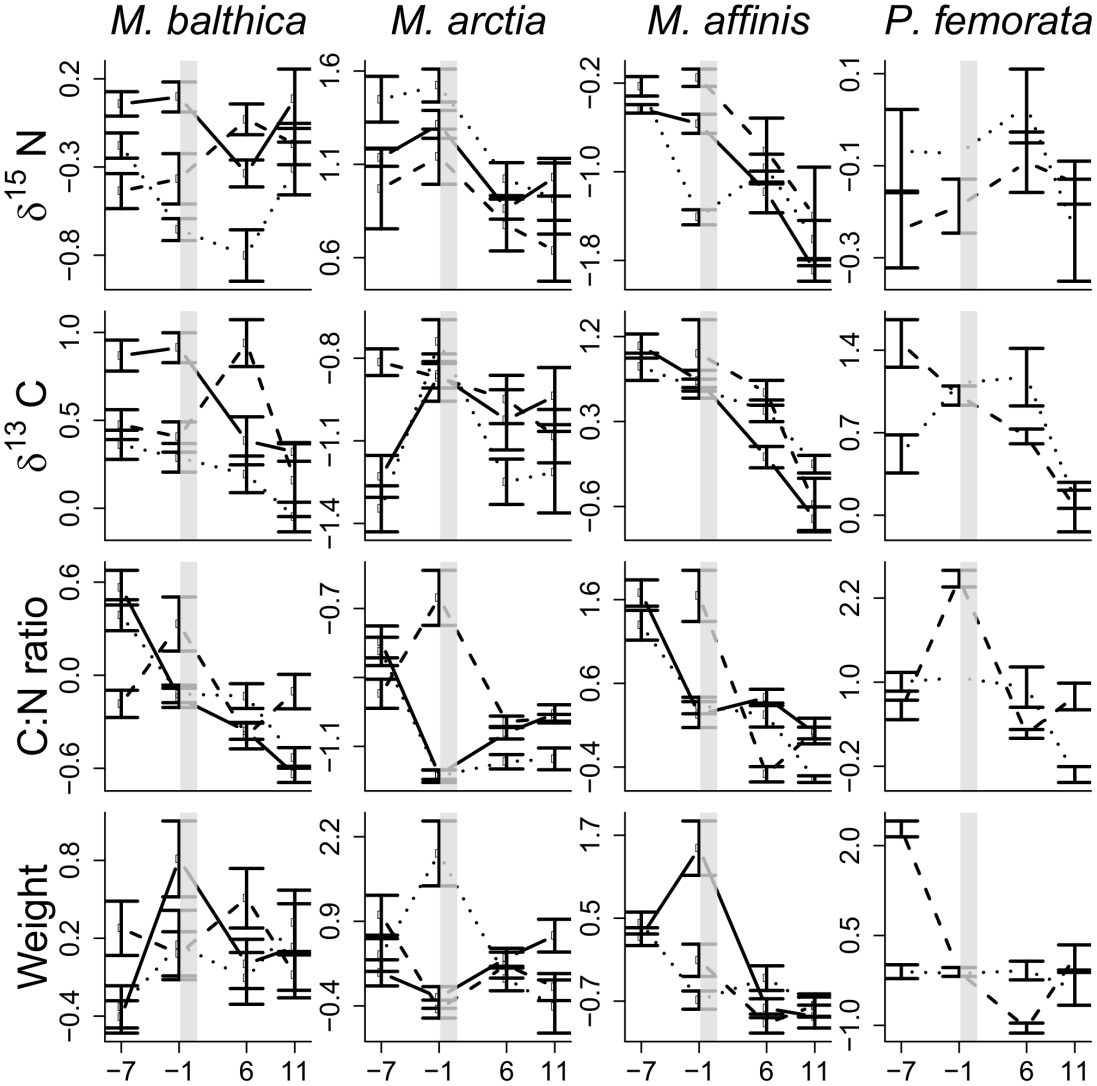
Change in faunal isotope and elemental composition during summer. Temporal trends in δ^15^N, δ^13^C, C:N ratio and individual weight (top to bottom panels) for each species and station (solid line = stn Uttervik, broken line =  stn Mörkö and dotted =  stn Håldämman). The peak of the bloom is indicated by the grey bar. The pre-bloom samples were taken 7 and 1 weeks before the peak of the bloom and the post-bloom samples were taken 6 and 11 weeks after the bloom. Data are transformed to z-scores and pooled for 2009 and 2010. Error bars are 95% CI (n∼20 for each sampling occasion; see also Table 1).

Macrofauna was sampled with a benthic sled, set to collect the top 1–2 cm of sediment, which was sieved through a 1 mm mesh to retain the macrofauna. The two most abundant species, the Baltic clam *Macoma balthica* and the non-indigenous spionid polychaete *Marenzelleria* cf. *arctia* [37], were found at all stations on all sampling occasions. The amphipod *Monoporeia affinis* was less abundant and absent from stn Mörkö in May and July 2009. The amphipod *Pontoporeia femorata* was never found at stn Uttervik and absent from stn Håldämman in May and July 2009. On each sampling event ∼10 individuals of each species of approximately 2 mg dry weight (shell-free for *M. balthica*) were collected for isotope analyses. Due to the unbalanced sampling design and the different timing of the bloom between years and stations, we pooled the two pre-bloom sampling occasions and those after the bloom (in 2009) in the statistical analyses (Table 1, but see Figure 2). This simplified comparisons among stations and between years and facilitated the statistical niche analysis, which requires relatively large sample size.

**Table 1:** Number of individuals for each species (Mac = *Macoma balthica*, Mz = *Marenzelleria arctia*, Mon = *Monoporeia affinis* and Pon =  *Pontoporeia femorata*), station and year, used for stable isotope analysis.

	Uttervik				Håldämman				Mörkö			
	2009		2010		2009		2010		2009		2010	
	B	A	B	A	B	A	B	A	B	A	B	A
**Mac**	20	20	20	10	15	20	17	10	20	20	10	10
**Mz**	20	20	22	9	16	20	20	14	20	20	13	10
**Mon**	20	20	20	20	16	20	20	8	0	17	6	10
**Pon**	0	0	0	0	0	20	20	10	10	16	10	15

B denotes samples taken before the cyanobacteria bloom, A those after the bloom; zero - not found.

### Stable Isotope Analyses

Animals and bulk sediment (upper 1–2 cm, from the sled) from each sampling occasion were oven-dried (60°C), packed individually into tin capsules and analysed for elemental and stable isotope content (carbon and nitrogen) at the UC Davis Stable Isotope Facility, USA. The C and N isotope ratios are expressed in the ‰ notation, using the equation:where R is the ratio between the heavy and light isotopes (^13^C:^12^C or ^15^N:^14^N). The stable isotope ratio, denoted by δ, is defined as the deviation in ‰ from the international reference standard (Vienna PeeDee Belemnite for C, and atmospheric nitrogen gas for N). Higher δ indicates a higher proportion of the heavy isotope. Samples were run in continuous flow with a standard deviation of <0.2‰ among replicate standard samples both for C and N. The δ^13^C values in animals were corrected for lipid content according to Post et al. [38]. Rawdata on isotope and elemental composition in benthic fauna can be found in Table S1.

### Data Analyses And Statistics

#### Biomass And Body Condition

The body condition was assayed as C:N ratio (w/w), with higher values reflecting higher fat content [39]. All species were tested for possible correlation between C:N ratio and weight (Pearson product moment correlation) for each station and year, before and after the bloom. Only for *M. affinis* at stn Uttervik in 2009 and stn Mörkö in 2010, was it necessary to adjust C:N ratio for reduced individual weight: where A is the regression coefficient for the significant weight-C:N relationship; this adjustment is mathematically equivalent to using the residual C:N values of the weight-C:N regression. Adjusted values are used in the figures and all statistical tests, with the exception of the statistical models for isotope data, where raw data were used as explanatory variables for δ^15^N.

For each species, pre-bloom and post-bloom values for individual body weight and C:N ratio were compared using paired t-tests with station as grouping factor. Pearson correlations and t-tests were performed in STATISTICA 12 (StatSoft).

#### Generalized Linear Models

Markedly higher δ^15^N values were observed at stn Mörkö, due to the nearby sewage treatment plant [40],[41]. Since differences among the stations were not the focus of our study, all data (δ^15^N, δ^13^C, C:N ratio, weight) were normalized to z-scores (zero mean, unit variance) for each station and year to remove the baseline differences in population means between samples collected in different years and at different stations, and to bring the distributions closer to normal.

The effect of the bloom on δ^15^N in the test populations was tested separately for each species in generalized linear models using the GLZ module in STATISTICA 12 (Statsoft) with a log-link error structure and AIC (Akaike information criterion) as the selection criteria. The full model included all categorical variables; bloom (2 levels, before and after), station (3 levels) and year (2 levels), and the three continuous variables: body weight, C:N ratio and δ^13^C values. Body weight and C:N ratio are indicative of ontogeny and body condition, respectively, and δ^13^C correlated strongly to δ^15^N for most species, as frequently observed [26]. The effect of the bloom on the δ^13^C was tested using the same categorical factors as for δ^15^N, but with the body weight as the single continuous variable. The response variables, δ^15^N and δ^13^C, were Box-Cox transformed prior to analyses. Goodness of fit and residuals were examined to confirm that the selected error distribution and link function were appropriate. Two outliers for *M. affinis* and *M. arctia*, respectively, were identified using Grubbs test and removed prior to the analyses. This removal did not affect the analyses outcome but improved normality of residuals.

#### Niche Metric Calculations And Statistics

Isotope niche metrics [33],[34] were calculated for each species, station and year, before and after the bloom, using the SIBER (Stable Isotope Bayesian Ellipses in R) package for R v.2.10.135 [42], included in the package SIAR [43]. The metrics developed by Layman et al. [33] represent different aspects of trophic diversity. A large δ^15^N range (dN) indicates greater trophic diversity in terms of the trophic position and potential for omnivory, whereas a large δ^13^C range (dC) indicates multiple carbon resources (e.g., benthic, pelagic, terrestrial runoff) and potential for niche diversification. Mean nearest neighbour distance (MNND) describes how individuals are distributed relative to one another within the dietary niche space of the population or the community, with higher values indicating greater dietary divergence. The standard ellipse area, SEA or SEAc when corrected for small sample size [34] provides information about the core aspects of a group (population or community) niche and can also be used to calculate niche overlap between species.

These niche metrics were calculated both for the populations in question (for each species, station, year, before and after bloom) and for the entire community at the same spatio-temporal resolution. Bloom effects on the niche metrics were tested using paired t-tests, with stations as a grouping factor, or Wilcoxon matched pair tests when assumptions of normality were not met. The relationship between the overlap area between the species sharing the same niche and the community niche area was tested using Spearman rank correlation.

#### Linking Niche Metrics To Bloom Intensity, Body Condition And Inter-Specific Competition

Bloom intensity was calculated as the area under the curve for the total cyanobacteria biomass from the mid May to the end of September using linear spline interpolation (XLfit, Microsoft Excel add-in). The relative change in SEAc following the bloom event was used as a dependent variable in the linear regression with bloom intensity and species as independent variables. We used SEAc because it is less affected by outliers and small sample size [44],[45] than the other metrics.

The mean body C:N ratio was calculated for all species, stations, and years, before and after the bloom. The relative change in the C:N ratio after the bloom event was also used as a dependent variable in the linear regression with bloom intensity and species as the independent variables. Finally, the relative change in C:N ratio after the bloom event was used as a dependent variable in the linear regression with relative change in SEAc (post-bloom to pre-bloom ratio) and species as independent variables. The significance of the categorical factor was tested using a homogeneity-of-slopes model. The amphipod *Pontoporeia femorata* was not included in these analyses since we found no evidence for uptake of cyanobacterial nitrogen by this species. To examine whether there is an exploitative competition between *M. balthica* and *M. affinis* (i.e. the species that overlapped most often), we tested if their SEAc values change in concert using Pearson product moment correlation. Residuals were examined to confirm normality prior to the analyses.

## Results

### Bloom Development And Species Composition Of Cyanobacteria

At stn B1 in 2009, the bloom started in late June, peaking on June 30, while in 2010, the bloom peaked only in mid-August. The bloom at stn H3 occurred in mid-July in both years. Bloom intensity was highest at stn B1 in 2009, with 30–35% lower values observed at stn H3 (both years) and stn B in 2010. In 2009, *Aphanizomenon* constituted 76 and 78% of diazotrophic biomass at stn H3 and stn B1, in 2010 the contribution was higher; 82 (stn H3) and 85% (stn B1). *Nodularia spumigena* was the second most abundant species at stn B1 (15 and 11% in 2009 and 2010, respectively), whereas *Anabaena* spp. contributed more (16 and 13%) at stn H3. At both stations in 2007–2009, seston δ^15^N mirrored nitrogen fixation, declining from a spring bloom maximum to a minimum (2–4‰) two weeks after the peak of the cyanobacteria bloom (coinciding with the third sampling event in 2009), and then increasing again [46].

### Elemental Composition And Isotope Ratios Of Sediments And Test Species

Elemental content in the sediment varied from ∼3.5% C and 0.5% N at stn Mörkö to ∼5.5% C and 0.8% N at stn Uttervik, with intermediate values at stn Håldämman (Table 2). In 2009, sediment values of δ^15^N, C% and N% were lower after the bloom only at stn Uttervik, but in 2010 they were lower at all stations.

**Table 2:** Sediment element composition and isotope ratio before (B) and after (A) the bloom.

	Uttervik				Håldämman				Mörkö			
	2009		2010		2009		2010		2009		2010	
	B	A	B	A	B	A	B	A	B	A	B	A
**C%**	5.6	5.0	5.3	5.0	4.1	5.0	5.1	5.1	3.3	3.5	3.8	3.5
**N%**	0.86	0.72	0.72	0.67	0.64	0.77	0.73	0.72	0.46	0.49	0.51	0.46
**δ^13^C**	−22.4	−22.2	−22.7	−22.5	−22.6	−22.7	−23.0	−23.1	−23.3	−22.6	−23.0	−23.2
**δ^15^N**	4.68	4.40	5.22	4.80	3.77	4.40	5.22	4.75	5.23	5.95	6.61	5.95

Values are means based on 3–6 analytical replicates per station and time point. Precision was <0.1% for C and N and <0.1‰ for δ^13^C and δ^15^N.

**Figure 3:**
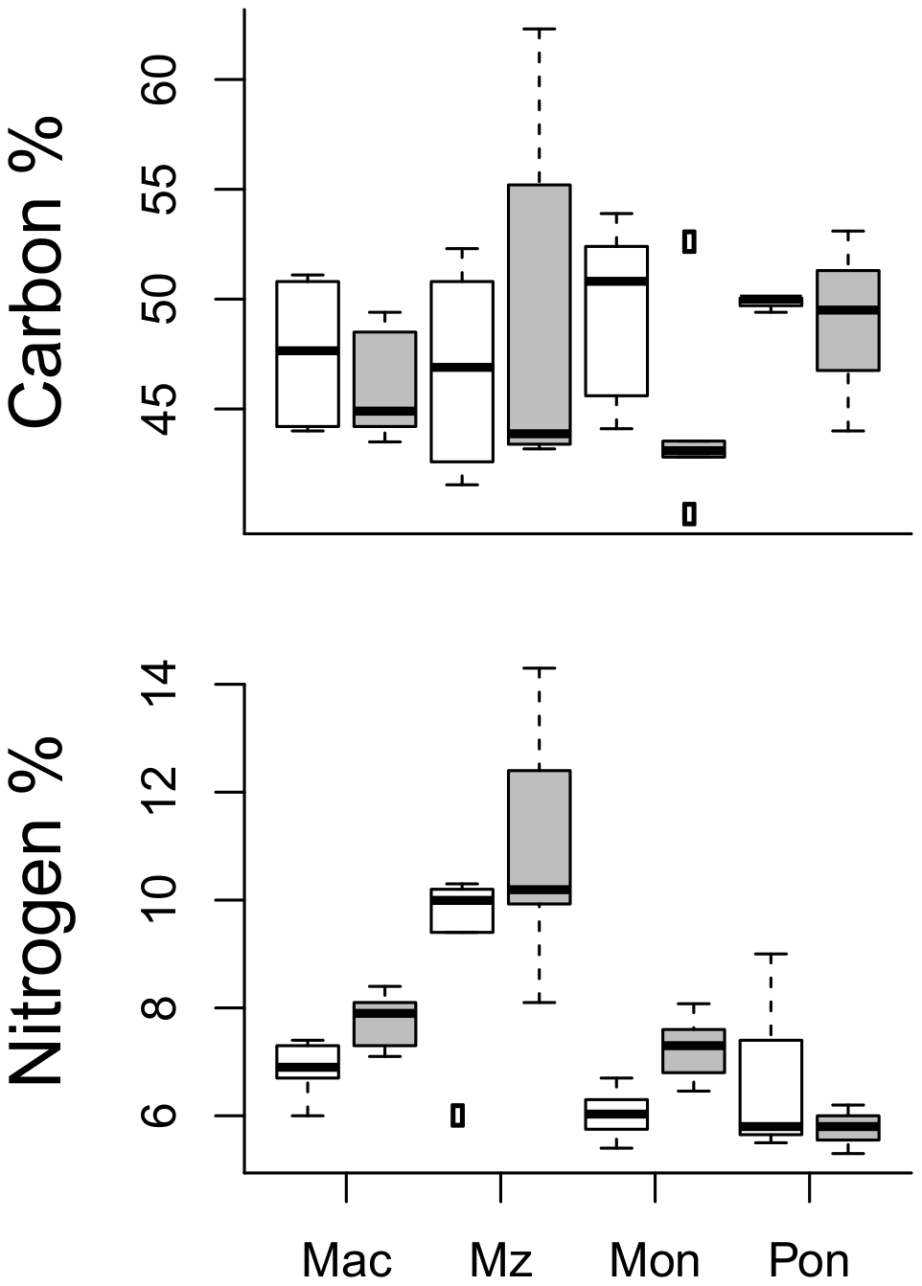
Carbon and nitrogen content pre- and post-bloom. Carbon and nitrogen (%) in the test species (left to right: Mac - *M. balthica*, Mz - *M. arctia*, Mon - *M. affinis* and Pon - *P. femorata*) before (white) and after (grey) the bloom. Values are median with 25 and 75% percentiles as well as 95% CI (n = 6 for Mac and Mz, n = 5 for Mon and n = 3 for Pon).

Macrofaunal body condition, indicated by the C:N ratio, changed over the season (Figure 2). In *M. affinis*, both individual weight and lower C:N ratio were lower after the bloom (Paired t-tests, biomass: t = 2.66, p = 0.057, C:N ratio t = 4.60, p = 0.01). In the other species, no change in the individual weight from the pre-bloom to the post-bloom were observed (*M. balthica* p = 0.82, *M. arctia* p = 0.53), but their C:N ratios declined (*M. balthica*, t = 3.41, p = 0.02, *M. arctia*, t = 2.11, p = 0.09). The small sample size for *P. femorata* precluded meaningful comparisons. Nitrogen content (%) increased after the bloom for the surface-feeding species (*M. balthica* and *M. arctia*: Z = 2.20, p = 0.028, n = 6, *M. affinis* Z = 2.02, p = 0.043, n = 5), with no change in C% (*M. balthica* Z = 1.15, p = 0.25, *M. arctia* Z = 0.94, p = 0.35, *M. affinis* Z = 1.75, p = 0.08). The carbon and nitrogen content for each species and period are shown in Figure 3.

### Bloom Effects On The Isotope Ratios Of The Test Species

**Figure 4:**
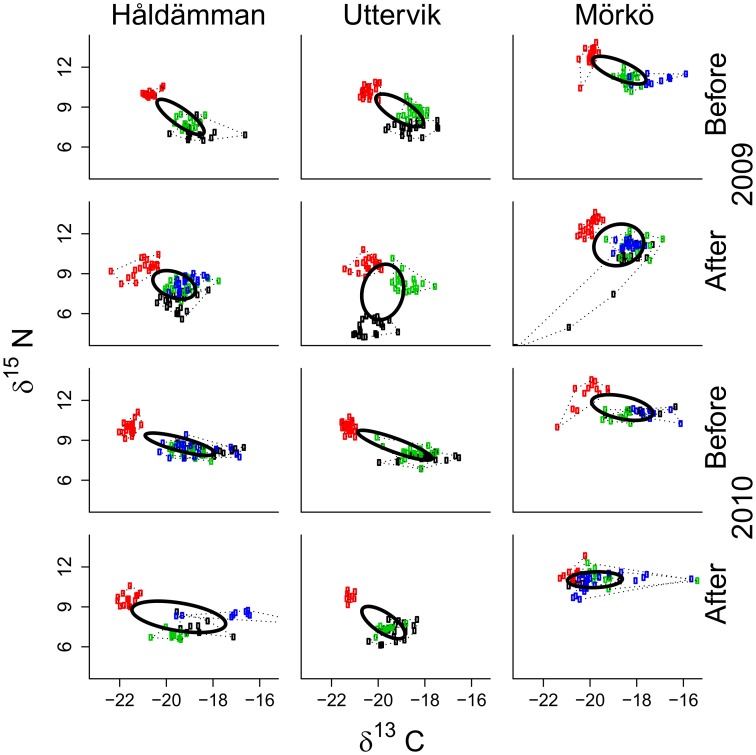
Isotopic niche for each species and the entire community pre- and post-bloom. Stable isotope bi-plots illustrating the isotopic niche of the four species, *M. affinis* (black), *M. arctia* (red), *M. balthica* (green), *P. femorata* (blue) at three study sites (top stn Håldämman, mid stn Uttervik and bottom stn Mörkö) before and after the bloom, for both years (2009, left panels, and 2010, right panel). The dotted lines enclose convex hull area for each species and solid line shows the standard ellipse area, SEAc, for the entire community. Note the presence of *M. affinis* post-bloom at stn Mörkö 2009 inflates the niche area, since it was not found pre-bloom. Excluding *M. affinis* at this station in 2009 results in a reduced niche area (as in Fig 5, top panel).

For all species except *P. femorata*, the δ^15^N values became significantly depleted after the bloom (Figure 2, Table 3). The δ^13^C values were also significantly depleted after the bloom for *M. balthica* and the amphipods, but not for *M. arctia* (Figure 4, Table 3). In line with these observations, bloom was a significant explanatory variable in all winning models, with the exception of δ^15^N for *P. femorata* (Table 3).

**Table 3:** GLM results for δ^15^N and δ^13^C for all species.

Model	Species	Variable	Estimate	SE	Wald stat	p
**δ^15^N**	***M. affinis***	Weight	0.11	0.02	38.70	<0.001
		δ^13^C	0.05	0.02	8.31	<0.01
		Bloom	−0.11	0.04	7.38	<0.01
**δ^13^C**	***M. affinis***	Bloom	−0.33	0.04	84.37	<0.001
		Mörkö	0.095	0.04	3.86	0.05
		Håldämman	−0.03	0.05	0.85	0.36
**δ^15^N**	***M. arctia***	δ^13^C	0.10	0.02	33.55	<0.001
		Bloom	−0.19	0.03	31.10	<0.001
**δ^13^C**	***M. arctia***	Bloom	−0.08	0.05	2.31	0.13
**δ^15^N**	***M. balthica***	C:N	−0.08	0.04	5.59	0.03
		δ^13^C	0.067	0.03	5.10	0.02
		Bloom	−0.15	0.05	9.00	<0.01
**δ^13^C**	***M. balthica***	Bloom	−0.19	0.05	14.58	<0.001
**δ^15^N**	***P. femorata***	Weight	0.11	0.05	5.46	0.02
		C:N	−0.11	0.05	5.02	0.03
**δ^13^C**	***P. femorata***	Bloom	−0.22	0.11	4.21	0.04

Only winning models according to AIC criteria are shown. The reference category for the estimate for bloom is the pre-bloom; negative values denote a decrease after bloom. The reference station is stn Uttervik.

### Population Level Niche Metrics And Bloom Intensity, Competition And Faunal C:n Ratio

**Figure 5:**
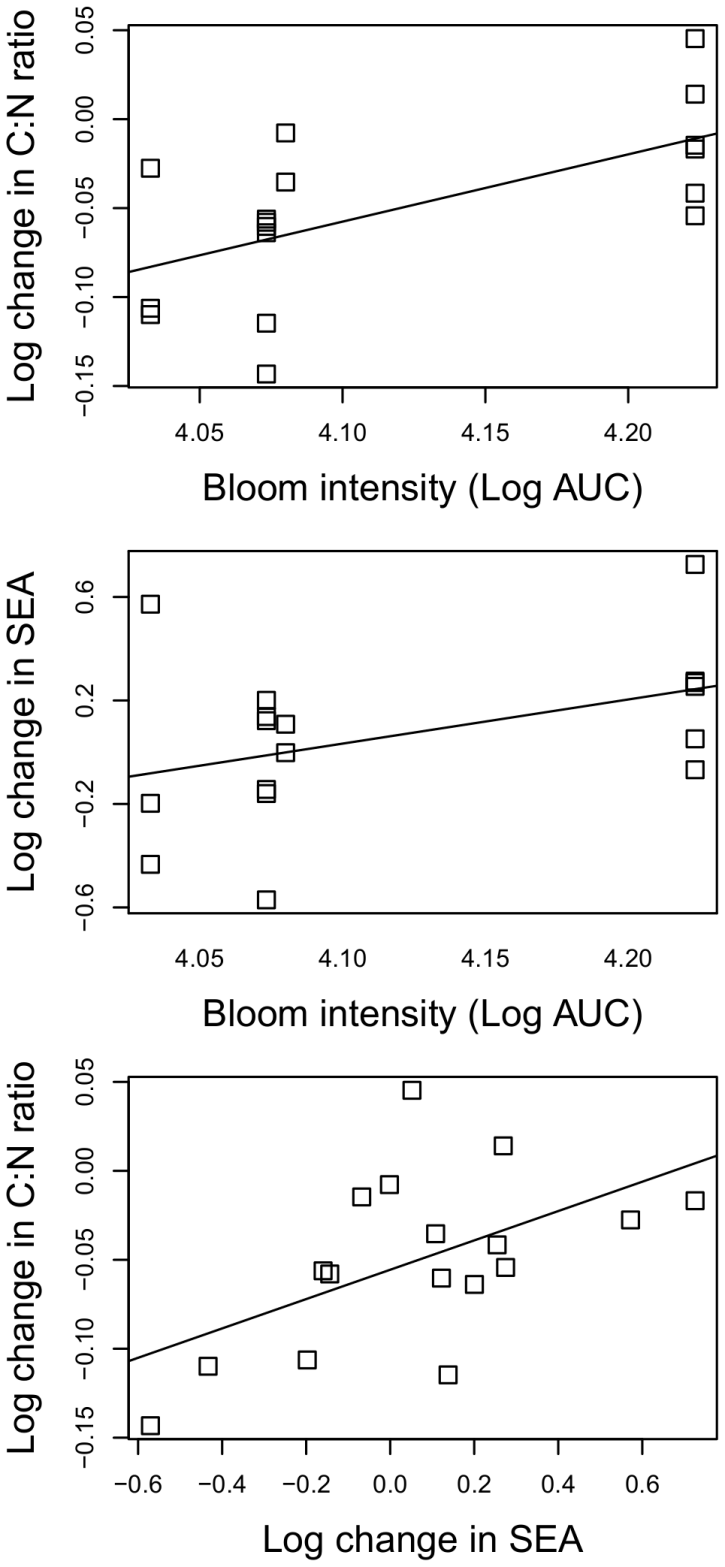
Relationship between bloom intensity and bloom induced change in C:N and isotopic niche. Concurrent changes (post-bloom/pre-bloom) in C:N ratio and SEAc in relation to the bloom intensity (area under curve; top and mid panels) and to each other (bottom panel). Each data point represents one species, station and year.

Testing for a bloom effect across all niche metrics and species results in 12 tests, and when applying a Bonferroni corrected critical p-value, no test was significant. This was due to large variability between stations and years and the small sample size (n = 5 or 6). A significant negative correlation in SEAc change after the bloom was observed between *M. affinis* and *M. balthica* (Pearson product moment correlation r = −0.89, p = 0.04). The post-bloom to pre-bloom SEAc ratio explained 25% of the variation in the post- to pre-bloom C:N ratio (F_1,15_ = 6.42 p = 0.02, adj r^2^ = 0.25) - when pooling the three species that were found to respond to the cyanobacteria bloom (Figure 5). When *M. affinis*, which appeared to differ from the other two species, was removed from the model, the SEAc explained 54% of the C:N change (F_1,10_ = 14.12, p<0.01, adj r^2^ = 0.54). Bloom intensity explained 32% of the variation in C:N ratio change following the bloom (F_1,13_ = 9.84 p<0.01, adj r^2^ = 0.32), with no significant species effect (p = 0.68, Figure 5a). Bloom intensity explained little variation in SEAc change after the bloom (F_1,15_ = 3.65, p = 0.07, adj r^2^ = 0.14; Figure 5b).

### Niche Overlaps And Community Niche Pre- And Post-Bloom

**Figure 6:**
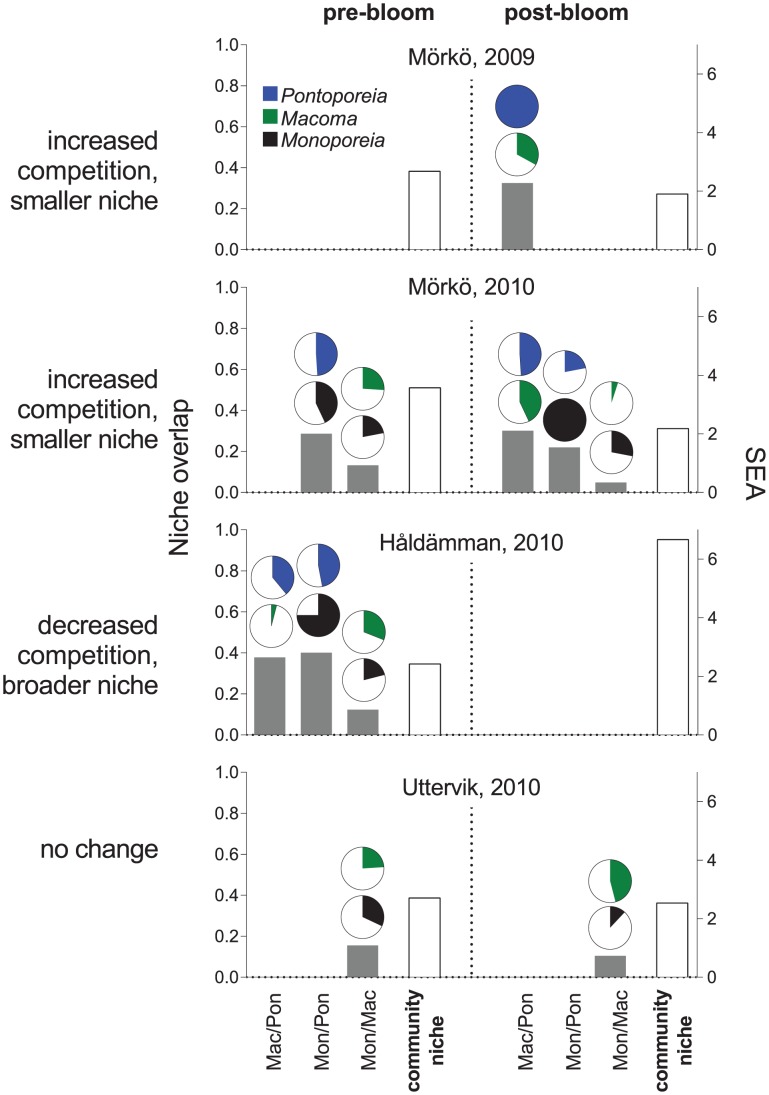
Cross-species overlap in isotopic niche pre- and post-bloom. Isotopic niche overlap defined as an area common for the two populations in relation to the total isotopic space occupied by these populations (proportion; left y-axis) and total community niche (arbitrary units; right y-axis) calculated using pre-bloom and post-bloom datasets for each station and year (see also Fig. 4 for raw data). The grey bars show the overlap estimate for each pair of species whose niches were found to overlap, white bars are the total community niche, and pie charts on the top of the gray bars show overlapping proportions of the isotopic niche for each population in question. Datasets that have no overlap during the study period are not included.

The isotope niche of *M. arctia* was distinct from the other species (Figure 4), but overlapped slightly (∼4% of the total niche area) with *M. balthica* on one occasion, after the 2010 bloom at stn Mörkö. For species that were found at a station before and after the bloom, the amount of overlap correlated negatively with the SEAc (Spearman R = −0.88, t = −5.28, p<0.01, n = 10). As such, post-bloom overlaps were recorded at stations that showed a decreasing or unchanged SEAc after the bloom, but not where the SEAc increased (Figure 6). At the stations with no cross-species overlap (stns Uttervik 2009 and Håldämman 2009) SEAc also increased considerably after the bloom (112 and 32% increase, respectively). Moreover, stns Uttervik and Håldämman showed increased dN range (56–19% and 6–9% increase) and MNND (25–34% and 37–100% increase) in 2009 and 2010 respectively, whereas stn Mörkö had nearly unchanged dN range, 29 and 39% reduction in SEAc and 9 and 29% lower MNND after the bloom in 2009 and 2010, respectively.

## Discussion

We found clear evidence that diazotrophic nitrogen derived from the summer cyanobacteria bloom is incorporated into the benthic food webs in the Baltic Sea, with significant differences among the test species. As hypothesized, depleted δ^15^N values were found in *Monoporeia affinis*, *Macoma balthica* and *Marenzelleria* cf. *arctia* after the bloom (Figure 2). These species are all surface-feeders, while *Pontoporeia femorata*, which did not show depleted values after the bloom, is primarily a sub-surface feeder [9]. In line with this, the observed lack of response in *P. femorata* likely reflects its lower use of bloom material newly settled on the sediment surface. The changes in carbon and nitrogen content in the test species provide supporting evidence for this explanation: all species, except *P. femorata*, increased their nitrogen content (%) without significant loss in C% (Figure 3). This increase in N% most likely reflected usage of cyanobacterial matter with relatively high nitrogen content and beneficial amino-acid composition [20]. All species also had more depleted carbon isotope values after the bloom, as hypothesized. The observed depletion in carbon values may, however, be also caused by uptake of other than of cyanobacterial C, e.g., aged sediment, which is more depleted in δ^13^C than seston [47], as a result of microbial activity [48]. Therefore, the observed decrease in δ^13^C of *P. femorata*, but unchanged δ^15^N, may reflect feeding on aged sediment rather than cyanobacteria.

Variations in isotopic fractionation can also contribute to the seasonal changes in isotopic signatures of consumers [49]. The depleted δ^15^N signal in benthic fauna after the bloom is, however, unlikely to be confounded by variation in fractionation. Despite the cyanobacterial input of nitrogen, the availability of nitrogen decreased during summer (Table 2). This should result in a lower growth rate (as is indeed the case for amphipods, [13],[36], and, hence, higher fractionation of ^15^N [49]. Microbial conditioning of the cyanobacterial bloom material can also increase δ^15^N values [50], complicating pre- and post-bloom comparisons of the isotopic signatures in bulk sediment samples. Hence, two main opposing processes may influence sediment and faunal δ^15^N values: (1) settling and incorporation of diazotrophically fixed nitrogen, which results in depleted δ^15^N values, and (2) trophic turnover in sediment microbial loops, which results in enriched δ^15^N values. Despite the latter process, the δ^15^N values in the key macrofauna benthic species became significantly depleted after the bloom.

Isotopic signatures do not, however, prove direct feeding on cyanobacterial filaments. Much of the diazotrophic nitrogen incorporated in the benthic fauna may have passed through other phytoplankton in the water column or through heterotrophic bacteria, both pelagic and benthic. A large proportion of fixed dinitrogen is known to leak from the cell as ammonium or dissolved organic nitrogen that can be assimilated by other phyto- and bacterioplankton [51]. Using isotope tracers, incorporation of cyanobacterial nitrogen by macrofauna has been estimated to contribute 5–10% of the animal nitrogen content [11]. Cyanobacteria have also been estimated to contribute significantly to zooplankton nitrogen content [27],[52]. On shallow sandy bottoms, Nordström et al. [28] found δ^15^N values depleted by several ‰ in late summer at all trophic levels studied; macroalgae, macrobenthos and fish. Similarly, Lesutiene et al. [29] found depleted δ^15^N after the cyanobacteria bloom throughout the shallow Curonian Lagoon food web, and estimated that diazotrophically fixed nitrogen may support up to 80% of secondary production during the bloom period. Interestingly, our most depleted values were also found at the shallowest station, indicating a stronger influence of cyanobacteria in these habitats. In shallow waters, the animals have generally higher growth rates (*M. affinis*: [36]), and higher incorporation rates would thus be expected. Taken together, these findings indicate that cyanobacterial blooms are likely to be important for secondary production of all benthic communities during summer, and particularly so in shallow habitats.

Our hypothesis that the niche area would increase as a result of diversified food supply after the bloom, which – at least to some degree – could alleviate nutrient limitation, found only partial support (Figures 4, 6). These responses appear to be complicated by the magnitude of bloom, sediment nutrient content and inter-specific competition. The community-level responses included reduced overlap between the species along with an increase in niche area (SEAc) following the bloom (Figure 6). This was further supported by increased MNND at stations Uttervik and Håldämman (both years) implying that, although no overlap was found throughout 2009, the dietary divergence between individuals increased after the bloom. However, the pattern was different at stn Mörkö, where we observed reduced SEAc and MNND and increased isotopic niche-area overlap between all species (Figure 6). Mörkö had the lowest sedimentary C and N content (Table 2). One can speculate that, due to food limitation, the settling bloom material might be more actively utilized by all animals, resulting in higher niche overlap throughout the season.

Interestingly, the isotopic niche of the non-indigenous species *M. arctia* was clearly different from those of potentially competing native species in all data sets, supporting previous experimental studies demonstrating resource partitioning in the invaded communities [53]. Moreover, *M. balthica* and *M. affinis* are known to compete for fresh phytodetritus [53] and seldom co-occur in high numbers [54]. The inverse relationship between the bloom-induced change in their niche sizes provides supporting evidence for interspecific food competition, highlighting the importance of detailed knowledge on trophic interactions when interpreting isotopic niche.

At the population level, the increase in isotopic niche following the bloom event was positively related to change in C:N ratio. Similarly, when the cyanobacterial bloom was large, we observed less reduction in the C:N ratio than after a bloom of lower intensity (Figure 5a). All species, including *P. femorata*, which did not utilize diazotrophic nitrogen, showed reduced C:N ratio at the end of the summer. This pattern was expected for the amphipods, especially *M. affinis*, which has its main growth period after the spring diatom bloom [1],[13],[39], with growth rate, body condition and lipid stores normally declining during summer [36],[39]. Amphipods need to build up lipid reserves before the winter and it is possible that they can benefit from cyanobacterial matter only after pre-conditioning (trophic upgrading [23]) for longer than the duration of this study or published experiments [16],[17]. This could, at least partially, explain the stronger relationship between SEAc and C:N ratio when *M. affinis* was excluded from the analysis. Populations that increased their isotopic niche throughout the season (i.e. increased generalist feeding) had a better body condition than populations with unchanged or decreasing niches, suggesting that supplemental feeding may convey fitness advantages, which is rarely documented (but see [55]).

In conclusion, the incorporation of the bloom material into the food webs, as evident from the depleted δ^15^N signals in fauna, is advantageous for these species during the critical summer season. The possible role of cyanobacteria in supporting not only primary, but also secondary production in the Baltic Sea deserves more attention.

## Supporting Information

Table S1 Isotope and elemental composition (carbon and nitrogen) for each individual sampled. (XLSX)
